# Redefining Non-Inferiority in Anamnestic Antibody Responses Using the Mean Increase of Log-Transformed Antibody Titers after Revaccination: Secondary Analysis of a Randomized Controlled Rabies Vaccination Trial

**DOI:** 10.3390/vaccines8040721

**Published:** 2020-12-02

**Authors:** Lisanne A. Overduin, Patrick H. P. Soentjens, Jelle J. Goeman, Magdalena A. Berkowska, Jacques J. M. van Dongen, Leo G. Visser

**Affiliations:** 1 Department of Immunology, Leiden University Medical Centre (LUMC), 2333 ZA Leiden, The Netherlands; M.A.Berkowska@lumc.nl (M.A.B.); J.J.M.van_Dongen@lumc.nl (J.J.M.v.D.); 2 Department of Infectious Diseases, Leiden University Medical Centre (LUMC), 2333 ZA Leiden, The Netherlands; L.G.Visser@lumc.nl; 3 Department of Clinical Sciences, Institute of Tropical Medicine, 2000 Antwerp, Belgium; psoentjens@itg.be; 4 Centre for Infectious Diseases, Queen Astrid Military Hospital, 1120 Brussels, Belgium; 5 Department of Biomedical Data Sciences, Leiden University Medical Centre (LUMC), 2333 ZA Leiden, The Netherlands; J.J.Goeman@lumc.nl

**Keywords:** rabies vaccines, neutralizing antibodies, revaccination, boostability, immunogenicity, immunologic memory, pre-exposure vaccination, non-inferiority, intradermal injection

## Abstract

Non-inferiority in the anamnestic antibody response is conventionally determined by comparing seroconversion rates after revaccination. However, this approach is inadequate in the case of high pre-booster antibody titers. Therefore, we propose an alternative method to determine non-inferiority of booster responses. We used anonymized data from a randomized controlled trial (NCT01388985; EudraCT 2011-001612-62) in 500 adults, comparing a two-visit primary vaccination schedule (two intradermal 0.1 mL rabies vaccine doses on day 0 and 7) with a three-visit schedule (single intradermal 0.1 mL dose on day 0, 7, and 28). Participants were revaccinated intradermally (single dose) 1 to 3 years later. Rabies virus neutralizing antibody titers were measured on day 0 and 7 after revaccination. After log_3_-transformation of antibody titers, the mean increase in titers after revaccination was compared between schedules. Non-inferiority was defined as the lower bound of the two-sided 95% confidence interval not exceeding −0.369. Four hundred and ten participants fulfilled the inclusion criteria. The mean increase in log_3_ titer was 2.21 and 2.31 for the two-visit and three-visit schedule, respectively. The difference between these increases was −0.10 [−0.28, 0.08], meeting the non-inferiority criterion. In conclusion, comparing mean increases in log-transformed titers after revaccination appears to be a feasible and more informative method of studying non-inferiority regarding the anamnestic antibody response.

## 1. Introduction

The main purpose of vaccination is to induce a robust immunological memory response that can be addressed by revaccination after possible exposure to the antigen. Yet, the definition of such an adequate anamnestic immune response, or ‘boostability’, is unclear. For rabies vaccination studies, the rabies virus neutralizing antibody (RVNA) titers on 7 or 14 days after revaccination are often used as markers for boostability, as they directly correspond to the memory B-cell response to revaccination [1,2,3,4,5]. This anamnestic antibody response is primarily responsible for the protective immunity against infection with rabies virus, and it is therefore vital to be able to establish whether a memory response is adequate for protection.

Rabies virus is a lyssavirus that causes a fatal encephalitis upon infection [6]. Transmission through infected dog bites is the main cause (99%) of the 59,000 annual human rabies deaths, occurring mainly in Africa and Asia [7,8]. Fortunately, adequate prophylaxis by means of vaccination is available. In addition to thorough wound cleansing, post-exposure treatment in individuals without pre-exposure prophylaxis usually consists of local infiltration of the bite wound with anti-rabies immunoglobulins and the administration of four intramuscular rabies vaccine doses over a 3-week period [8]. If the exposed person has previously received pre-exposure prophylaxis, revaccination with two (intradermal or intramuscular) doses of rabies vaccine is sufficient [8].

Pre-exposure prophylaxis can be administered according to one of the two schedules as recommended by the World Health Organization (WHO): on both day 0 and 7, either (1) a single intramuscular vaccination with a full dose (1 mL) or (2) two intradermal vaccinations with a 0.1 mL fractional dose [8]. An adequate antibody response to pre-exposure rabies prophylaxis is generally defined as an RVNA titer of ≥0.5 IU/mL 21 to 28 days after primary vaccination [9,10].

Conventionally, an adequate anamnestic antibody response (referred to as ‘boostability’) is defined as the proportion of people who have a certain minimum antibody titer after revaccination (seroconversion rate, or SCR). As explained above, for rabies revaccination, this threshold for seroconversion lies at 0.5 IU/mL. If the majority of participants in a revaccination study have an antibody titer already exceeding this threshold at the moment of revaccination, their boostability cannot be adequately established, as they already reached the seroconversion endpoint before revaccination. This is problematic, especially when different pre-exposure vaccination schedules are to be compared on their respective boostability using this parameter. A recent systematic review of 36 studies on boostability after rabies revaccination provides a striking illustration of the problem at hand [11]. The seroconversion endpoint was already reached before revaccination in 67% of all intradermally and 90% of all intramuscularly vaccinated participants. The post-booster SCR was 99% or 100% for all schedules and studies, making it impossible to compare different vaccination schedules or administration routes based on this parameter alone. An option would be to increase the threshold to, for example, 10 IU/mL, but that does not solve the fundamental underlying problem of (high) pre-booster titers interfering with a fixed serological endpoint. Moreover, the clinical relevance of the threshold—the inferred minimum for protection against the antigen—disappears, and the outcome remains categorical and not continuous.

An alternative strategy that circumvents this problem is to compare the slope, or fold increase, of post-booster RVNA geometric mean titers between different vaccination schedules on day 7. A major advantage of this strategy is that it takes the pre-booster titer values into account. Furthermore, this method can be applied over a large range of antibody titers and contains more information in its assessment, as it is continuous and not categorical [12,13,14]. It might prove to be a suitable parameter to measure and compare boostability, in addition to the clinically relevant, yet difficult to compare between schedules, post-booster SCR. The proposed strategy is widely applicable and is also transferrable to revaccination studies with other vaccine antigens.

In this study, we evaluated the feasibility of such a quantitative approach to define boostability. For this purpose, we re-analyzed data from a previously conducted randomized trial that demonstrated non-inferiority using the post-booster SCR as primary endpoint [2]. We calculated the mean difference between individual log_3_ pre- and post-booster titers for two different rabies pre-exposure vaccination schemes and compared these two mean differences to show non-inferiority of these two vaccination schedules regarding boostability.

## 2. Materials and Methods

### 2.1. Study Design

An anonymized dataset from a previous randomized controlled trial (NCT01388985; EudraCT 2011-001612-62; approved by the local ethics committee (protocol ITMC0211); October 2011–January 2016) was provided by the Institute for Tropical Medicine in Antwerp, Belgium. For details of the study design, we refer to the original paper [2]. In short, participants receiving pre-exposure rabies prophylaxis were randomized to one of two study arms. The control group received the—at that time—standard three-visit schedule of intradermal vaccination of a single fractional dose of 0.1 mL human diploid cell culture vaccine (HDCV) (day 0, 7, and 28). The intervention group received a shortened two-visit schedule with two intradermal doses of 0.1 mL HDCV (day 0 and 7). After one to three years, participants were revaccinated with a single intradermal vaccination of 0.1 mL HDCV. RVNA titers were measured on day 0 and day 7 after revaccination, using the rapid fluorescent focus inhibition test (RFFIT), which uses threefold dilution steps [15,16,17]. Values were reported as RVNA concentrations in IU/mL. In this paper, we will use ‘RVNA titers’ to refer to these values. The original paper demonstrated non-inferiority of the two-visit schedule to the three-visit schedule regarding boostability, using the post-booster SCR (proportion of participants having an RVNA titer ≥0.5 IU/mL on day 7 after revaccination) as primary endpoint.

### 2.2. Study Population

Participants, aged 18–47 years, were recruited from the Belgian Armed Forces. All subjects gave their informed consent for inclusion before they participated in the study. They were excluded if they had previously received rabies vaccinations, if they were pregnant or breastfeeding, or if they had a known or suspected immunodeficiency, chronic disease, mefloquine prophylaxis, or a known allergy to any of the vaccine components. They were also excluded if they would be in overseas deployment within 35 days after the first vaccination.

For the current study, a modified intention-to-treat analysis was performed. Participants were included if RVNA titers on both day 0 and day 7 after revaccination were documented. They were excluded if their RVNA titer on day 0 of primary vaccination was ≥0.5 IU/mL, and if they received the booster vaccination more than 3 years (>1095 days) after their first rabies vaccination.

### 2.3. Variables

The dataset contained anonymized data on the allocated study group, RVNA serology measurements on day 0 and day 35 after primary vaccination and on day 0 and day 7 after revaccination, and the exact days on which these titers were measured. The difference in log_3_ RVNA titers between day 0 and 7 after revaccination was the primary outcome variable.

### 2.4. Bias

As the data were generated in a randomized controlled trial, no confounding bias was expected.

### 2.5. Statistical Methods

RVNA titer values were transformed to log_3_ [titer] values to ensure a normal distribution of values and to narrow the range [18]. A logarithm base of 3 was chosen because of the threefold dilution steps in the RFFIT. Log transformation allows for easy and straightforward reconversion to non-log-transformed values, as the arithmetic mean of log-transformed values corresponds to the log geometric mean of the original values [19].

The mean and standard deviation of log_3_-transformed pre- and post-booster RVNA titers on day 0 and day 7 after revaccination were calculated for each allocated study arm. The individual differences between the log_3_ pre- and post-booster titers (log_3_ post-booster − log_3_ pre-booster = log_3_ (post-booster/pre-booster)) correspond to the fold increase (or slope) in log_3_ geometric mean titer. The individual differences in log_3_ titer were calculated and presented in a boxplot. The mean of these individual differences was calculated as well. Individual log_3_-transformed RVNA titers on day 0 and day 7 after revaccination were displayed on a scatterplot. To identify possible high- and low-/non-responders, a histogram of the differences in log_3_ titer was plotted for each schedule.

The geometric mean and standard deviation of non-log-transformed pre- and post-booster titers were calculated for each allocated study arm, as were the geometric mean and standard deviation of the individual differences in titer. For this calculation, negative values (i.e., a decrease in titer) had to be excluded, as geometric means cannot be calculated with negative values.

We assumed that the post-booster RVNA titers are not independent of the pre-booster RVNA titers. In case of baseline imbalance in pre-booster titers among the two schedules, an analysis of covariance (ANCOVA) would be carried out to calculate the mean differences in pre- and post-booster RVNA titers, adjusted for difference in baseline pre-booster values [20,21].

The difference between the mean log_3_ titer difference of the two-visit and three-visit schedule (mean_2-visit_ − mean_3-visit_) was reported with a two-sided 95% confidence interval (CI), as calculated with an independent *t*-test. If the lower bound of this confidence interval was not less than −0.369, the two-visit schedule was considered to be non-inferior to the three-visit vaccination schedule It should be noted that −0.369 corresponds to a difference of log_3_ 1.5, which equals a 1.5-fold dilution step in the RFFIT. We have chosen this cut-off point because we considered a difference of less than one dilution step to be clinically irrelevant: a 1.5-fold dilution equals only half a dilution step in a 3-fold dilution step RFFIT.

To account for differences in time after primary vaccination, two additional analyses were carried out. Linear regression analysis was performed for the time after primary vaccination and log_3_ pre-booster titers, and for the time after primary vaccination and log_3_ titer increase. Results for these analyses were displayed in scatterplots with linear regression lines per allocated group.

Statistical analysis was carried out using R 3.6.3.

## 3. Results

### 3.1. Study Population

500 participants were included for 1:1 randomization; 498 participants received at least one rabies vaccination. Titer values on day 0 or day 7 after booster vaccination were unavailable for 81 out of 498 participants (16%); 3 out of the 417 remaining participants were revaccinated more than 3 years after primary vaccination, and 4 out of the 414 remaining participants had a positive rabies serology before primary vaccination. Therefore, 410 out of 500 (82%) participants were included in this analysis: 200 participants had been randomized to the 3-visit schedule and 210 participants to the 2-visit schedule. For descriptive characteristics of the groups, we refer to the original paper [2].

### 3.2. RVNA Titer Distribution

The geometric means and geometric standard deviations of pre-booster and post-booster titers and the corresponding log_3_-transformed means and standard deviations are displayed in Table 1. For the three-visit schedule, pre-booster log_3_ RVNA titers (day 0 of revaccination) ranged from −1.67 to 3.97 (mean ± *SD*: 0.65 ± 1.07) and post-booster log_3_ titers (day 7) ranged from 0.20 to 5.12 (mean ± *SD*: 2.96 ± 0.92). For the two-visit schedule, pre-booster log_3_ titers ranged from −1.67 to 3.41 (mean ± *SD*: 1.10 ± 0.98) and post-booster log_3_ titers ranged from 1.00 to 5.30 (mean ± *SD*: 3.31 ± 0.77). Two subjects showed a decrease in titer: one subject of the three-visit schedule showed a decrease from 78.29 to 45.75, and one subject of the two-visit schedule showed a decrease from 4.26 to 3.14.

**Figure 1:**
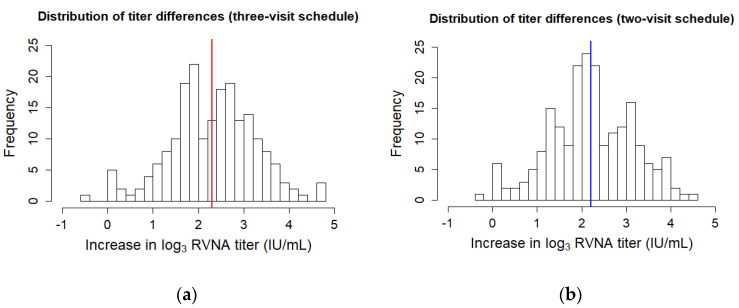
Histograms depicting the frequencies of the pre- and post-booster differences in log_3_ titer for the (**a**) three-visit schedule and (**b**) two-visit schedule. The mean difference is plotted as a thick line.

Histograms depicting the frequency of differences in log_3_ RVNA titer values show a normal distribution. There was no discrimination possible between high- and low-responders (Figure 1).

### 3.3. Non-Inferiority of the Mean Difference in Log Titers

**Figure 2:**
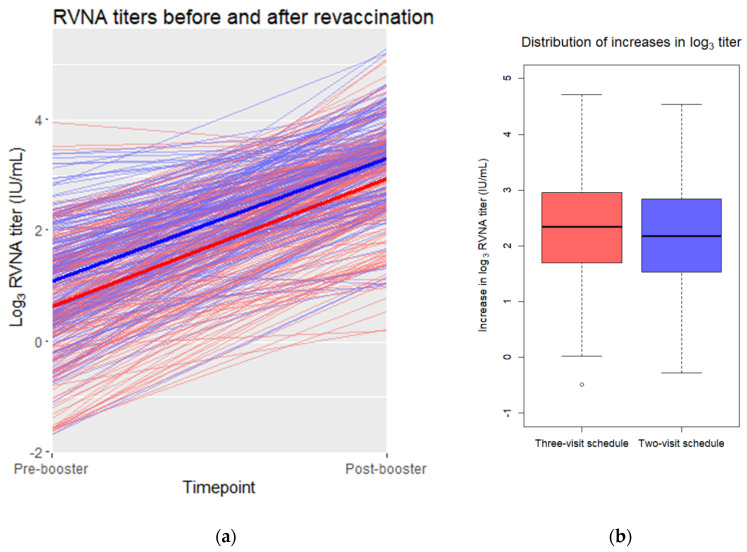
(**a**) Pre- and post-booster log_3_ RVNA titers in a scatterplot. Individual pre- and post-booster values are connected by thin red (three-visit schedule) or blue (two-visit schedule) lines. The mean values of the three-visit schedule are connected with a thick red line. The mean values of the two-visit schedule are connected with a thick blue line. (**b**) Distribution of the pre- and post-booster differences in log_3_ titer for each vaccination schedule. Depicted are the medians per schedule (horizontal line in the box) and the 25th and 75th percentile (upper and lower bound of the box). The whiskers represent the value in the data nearest to up to 1.5 times the interquartile range. One outlier, depicted as an open dot, falls outside of this range.

The unadjusted mean and standard deviation of the difference between log_3_ pre-booster and post-booster values was 2.31 ± 0.94 (range: −0.49 to 4.71) for the three-visit schedule and 2.21 ± 0.94 (range: −0.28 to 4.54) for the two-visit schedule (Figure 2). The difference between these unadjusted means was −0.10. An independent *t*-test provided a two-sided 95% confidence interval to this difference of [−0.28, 0.08]. The lower bound of the confidence interval did not exceed the non-inferiority margin of −0.369.

If non-log-transformed titer values were used (excluding one negative value from each study arm), the resulting unadjusted fold increase in geometric mean between pre-booster and post-booster values was 12.60 ± 2.80 for the three-visit schedule and 11.30 ± 2.81 for the two-visit schedule.

### 3.4. Baseline-Adjusted Non-Inferiority in the Mean Difference in Log Titers

**Figure 3:**
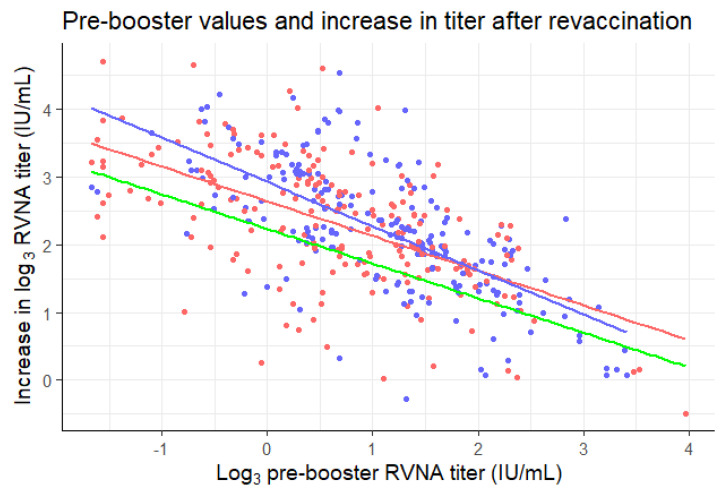
Pre-booster log_3_ titers plotted against the differences in pre- and post-booster log_3_ titers for the three-visit (red) and two-visit (blue) schedule. Linear regression lines were plotted for the data of each of the schedules in their corresponding color. Green line: three-visit schedule minus the non-inferiority margin of 0.369 (y = 2.6 − 0.51X − 0.369).

The mean of the log_3_-transformed pre-booster titers of the three-visit schedule was lower (0.65 ± 1.07) than the two-visit schedule (1.10 ± 0.98). To adjust for this baseline imbalance, an ANCOVA analysis would be suitable. However, the slopes of the regression lines of the groups were not homogeneous, as is shown in Figure 3. *R*^2^ values of the slopes were 0.34 and 0.47 for the three-visit and two-visit schedule, respectively. The slopes differed significantly between the allocated groups (*p* = 0.046). There were also three outliers (>3 standard deviations from the mean) in the data. This means that two of the assumptions necessary for ANCOVA were violated [22]. If the ANCOVA analysis was carried out despite this violation of assumptions, the mean of the difference between log_3_ pre-booster and post-booster values would be estimated to be 2.17 ± 0.74 for the three-visit and 2.33 ± 0.74 for the two-visit schedule. The difference between these adjusted means is 0.16, with an independent *t*-test providing a two-sided 95% confidence interval of [0.02, 0.30]. The lower bound of the confidence interval did not exceed the non-inferiority margin of −0.369, similar to the confidence interval of the unadjusted mean difference.

A remarkable observation in Figure 3 is the fact that even though the blue line (two-visit schedule) crosses the red line (three-visit schedule), it never crosses the green line (three-visit schedule minus the non-inferiority margin of 0.369). If the blue line would have crossed the green line, one could assume that the two-visit schedule is inferior to the three-visit schedule from that log_3_ pre-booster onwards. This is not the case and therefore solidifies the non-inferiority of the two-visit schedule to the three-visit schedule.

### 3.5. Effect of Time after Primary Vaccination on Log Titer Increase

**Figure 4:**
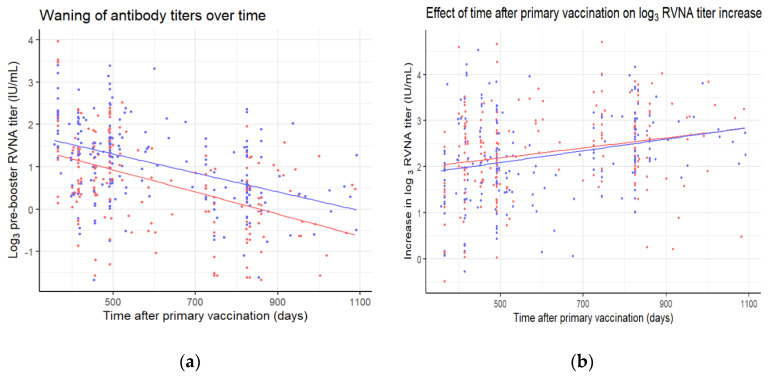
(**a**) Pre-booster log_3_ titers and (**b**) differences in pre- and post-booster log_3_ titers plotted against the time after primary vaccination for the three-visit (red) and two-visit (blue) schedule. Linear regression lines were plotted for the data of each of the schedules in their corresponding color.

Participants were revaccinated at different timepoints, between one to three years after primary vaccination. A longer time after primary vaccination is associated with lower log_3_ pre-booster titers (Figure 4a) (*p* < 0.001). The waning of antibodies is not significantly different between the allocated groups (*p* = 0.41). As can be seen in Figure 3, lower log_3_ pre-booster titers are associated with a higher log_3_ increase in titers (*p* < 0.001). Therefore, it is hardly surprising that a longer time after primary vaccination is associated with a higher log_3_ increase in titers as well (Figure 4b) (*p* < 0.001). This association does not differ significantly between the allocated groups (*p* = 0.68).

## 4. Discussion

In this study, we propose an alternative statistical method to determine non-inferiority in boostability in the case of high pre-booster antibody titers. We demonstrate the feasibility to infer non-inferiority in boostability by comparing the mean increase of log-transformed antibody titers after revaccination. The mean increase in log_3_ RVNA titers after booster vaccination was non-inferior for a shortened two-visit schedule compared to the standard three-visit schedule, confirming the results from the original paper [2].

A clear metric of the immunological memory response is a neglected topic in many studies. To assess immunological memory, studies need a long follow-up time, which is likely to lead to participants dropping out. On top of that, the memory response is hard to assess, as there is no clearly defined way of measurement. Assessing which proportion of a population has reached or exceeded a certain antibody titer is a flawed measure for the boostability of the immune system, especially in the case of high pre-booster baseline values and maximum post-booster seroconversion rates. For clinical purposes and at an individual level, the method makes perfect sense, because it is sufficient to know whether someone is protected or not. Unfortunately, in a research setting, this method is not suitable to infer non-inferiority when pre-booster proportions are already close to 100%.

We believe that the method proposed in this study has a major advantage compared to a proportional definition of boostability. A continuous variable maintains by definition more original input than a dichotomous proportional assessment. In the latter case, valuable information is lost due to a forced dichotomization [12]. For illustrative purposes: 88.5% of the three-visit schedule participants and 96.7% of the two-visit schedule participants in our data already had a pre-booster titer of 0.5 IU/mL or higher. With such a high proportion of individuals already at or above threshold for boostability before the actual revaccination, it becomes difficult to compare the two schedules on boostability using the 0.5 IU/mL threshold, especially considering that 100% of the participants of both schedules had a titer of 0.5 IU/mL or higher 7 days after revaccination, in which case they will always be non-inferior to each other. With this novel proposed method, non-inferiority in boostability—or the lack thereof—can be assessed in a more precise way.

This study has several strengths. Data from a large number of subjects from a randomized controlled trial was included in the analysis. The cut-off for non-inferiority was quite strict, as it was a difference of not more than a 1.5-fold dilution step between the means of the two groups, using a two-sided 95% confidence interval. Another, somewhat surprising, strength lies in the data in the form of the baseline imbalance in pre-booster titers. In general, such a baseline imbalance is in favor of an increase in the group with the lowest baseline values. The reason for this is that there is no linear relation between pre-booster and post-booster values, because the post-booster value has a certain maximum [23]. In this particular case, the three-visit schedule group had a lower mean baseline titer and therefore a greater mean increase in titer, if not adjusted. Yet, when adjustment for the baseline imbalance was performed, the mean increase for the two-visit schedule was higher than for the three-visit schedule. Unfortunately, not all assumptions for this analysis of covariance (ANCOVA) were met, so its results should be interpreted with caution. However, both the adjusted and unadjusted results clearly point to an overall non-inferiority of the two-visit schedule compared to the three-visit schedule.

From the previous paragraphs, it has become clear that demonstrating non-inferiority while using a proportional approach is hindered if pre-booster values are already high, and post-booster values approach or reach the maximum level (100% seroconversion). This is also the reason why superiority trials are generally not suitable for these kinds of research questions. A superiority trial with expected near-maximum post-booster levels would require an enormous sample size to significantly detect the very small difference in post-booster seroconversion rates (for example, a post-booster SCR of 99.7% versus 99.9%) [24,25,26]. However, our proposed numerical method could also be used in superiority trials, as it does not have the disadvantage of a maximum endpoint. In this way, novel, more efficient vaccination schedules can be studied on the topic of boostability with evidence for superiority rather than non-inferiority. However, one might still wonder whether superiority in boostability is a clinically and immunologically relevant endpoint. We would like to argue that it is immunologically relevant, as the results of the neutralization assay (RFFIT) reflect both the concentration and the affinity of the antibodies. As far as clinical relevance is concerned, being able to actually determine non-inferiority or superiority in boostability might form a stronger foundation for research on and implementation of simplified vaccination schedules in the future.

It is remarkable that the two-visit schedule seems to perform better regarding boostability than the three-visit schedule in individuals with low pre-booster values, but worse in individuals with higher pre-booster values (Figure 3). Furthermore, even though the blue line (two-visit schedule) crosses the red line (three-visit schedule), it never crosses the green line (three-visit schedule minus the non-inferiority margin of 0.369). This could be due to the formation of more memory B cells, or a longer lifespan of the germinal centers, in the two-visit schedule, during which participants received four 0.1 mL vaccine doses in one week, instead of ‘only’ three doses over three weeks [27]. Another interesting observation is the decrease in RVNA titer after booster vaccination in two cases. This seems highly improbable. It might be possible that this is a negative feedback mechanism at work, caused by the presence of pre-booster antibodies, which cause the antibody titer levels to drop [23]. This very same negative feedback mechanism might play a role in the observed lower increase for higher pre-booster antibody titers (Figure 3).

An important caveat in using the method as proposed in this study is the fact that we were not able to use a pre-existing cut-off point for non-inferiority. Therefore, we chose to define this point to correspond to a difference of a 1.5-fold dilution step. This cut-off point was chosen because we considered a difference of less than a half dilution step to be clinically irrelevant. A 1.5-fold dilution equals half of one 3-fold dilution step. The same cut-off point of a 1.5-fold dilution could also be applied in RFFIT assays using 2-, 10-, or any-fold dilution steps, although it does not equal half a dilution step in those assays. However, a 1.5-fold dilution denotes the same difference for all types of RFFIT: a 1.5-fold dilution step, regardless of the RFFIT method.

In future research on boostability, a different non-inferiority margin might be used, which may—also depending on the vaccine being studied—seem less or more reasonable than the 1.5-fold dilution step that we chose as the cut-off point. For example, Soonawala et al. used a 2-fold dilution step as the non-inferiority margin for the evaluation of the immunogenicity of a poliomyelitis vaccine [28].

Another choice for future research concerns the confidence interval. In this study, we opted for a two-sided 95% confidence interval, corresponding to a one-sided 97.5% confidence interval. However, one could also make a case for a two-sided 90% confidence interval (a one-sided 95% confidence interval), which increases the range over which non-inferiority can be inferred [26]. Still, the smaller the cut-off value and the broader the confidence interval, the stronger the evidence for non-inferiority. This emphasizes the importance of the documentation of a clearly defined and clinically relevant non-inferiority margin in the study protocol prior to data analysis [29].

For future studies, we propose using the more informative method of comparing the mean increase of log-transformed pre-booster antibody titers after revaccination next to the clinically relevant measurement of proportions. This method is easy to use, holds more (numerical) information than the proportional assessment, is applicable in superiority trials, and takes pre-booster values into account. Therefore, we advise researchers to consider using this alternative method as an additional outcome for measuring the boostability of the anamnestic antibody response.

## 5. Conclusions

In this study, it was demonstrated that it is feasible to use the mean increase of log-transformed antibody titers after revaccination as a marker for the anamnestic antibody response. It is a suitable alternative or addition to the conventionally used seroconversion rates, as this novel approach eliminates the main disadvantages of seroconversion rates. First of all, it takes pre-booster titers into account, instead of only post-booster titers. Secondly, it is more informative, as numerical information is retained in this parameter. Lastly, superiority trials regarding the anamnestic antibody response can be performed with this approach, whereas seroconversion rates often only allow for non-inferiority trials. Researchers are therefore encouraged to take this parameter in consideration when designing long-term vaccination studies. 

## Figures and Tables

**Table 1:** Mean rabies virus neutralizing antibody (RVNA) titer values for the different vaccination schedules before and after revaccination.

Type of RVNA Titer Measurement	Three-Visit Schedule		Two-Visit Schedule	
	Log_3_ Value	Non-Log-Transformed Value	Log_3_ Value	Non-Log-Transformed Value
Pre-booster (mean ± SD)	0.65 ± 1.07	2.04 ± 3.26	1.10 ± 0.98	3.34 ± 2.95
Post-booster (mean ± SD)	2.96 ± 0.92	25.74 ± 2.76	3.31 ± 0.77	37.77 ± 2.33
Difference between post-booster and pre-booster (mean ± SD) *	2.31 ± 0.94	12.60 ± 2.80	2.21 ± 0.94	11.30 ± 2.81

* For the log_3_ values, this row displays the additive difference between post-booster and pre-booster titers. For the non-log-transformed values, this row displays the multiplicative difference (fold increase) between post-booster and pre-booster titers.
